# Validation of a Device for the Ambulatory Monitoring of Sleep Patterns: A Pilot Study on Parkinson's Disease

**DOI:** 10.3389/fneur.2019.00356

**Published:** 2019-04-11

**Authors:** Carlos Javier Madrid-Navarro, Francisco Javier Puertas Cuesta, Francisco Escamilla-Sevilla, Manuel Campos, Fernando Ruiz Abellán, Maria Angeles Rol, Juan Antonio Madrid

**Affiliations:** ^1^Neurology Service, Hospital Universitario Virgen de las Nieves, Granada, Spain; ^2^Instituto de Investigación Biosanitaria ibs. GRANADA, Granada, Spain; ^3^Unidad de Sueño, Hospital Universitario de la Ribera de Alzira, Valencia, Spain; ^4^Centre de Sommeil, Service de Neurologie, CHU Liege, Liege, Belgium; ^5^Chronobiology Laboratory, IMIB-Arrixaca, CIBERFES, Instituto de Salud Carlos III, Universidad de Murcia, Murcia, Spain; ^6^Electronics Laboratory, SAI, University of Murcia, Murcia, Spain

**Keywords:** Parkinson's disease, sleep, circadian rhythms, ambulatory recordings, actigraphy, polysomnography, thermometry

## Abstract

The development of *wearable* devices has increase interest in the use of ambulatory methods to detect sleep disorders more objectively than those permitted by subjective scales evaluating sleep quality, while subjects maintain their usual lifestyle. This study aims to validate an ambulatory circadian monitoring (ACM) device for the detection of sleep and wake states and apply it to the evaluation of sleep quality in patients with Parkinson disease (PD). A polysomnographic validation study was conducted on a group of patients with different sleep disorders in a preliminary phase, followed by a pilot study to apply this methodology to PD patients. The ACM device makes it possible to estimate the main sleep parameters very accurately, as demonstrated by: (a) the lack of significant differences between the mean values detected by PSG and ACM in time in bed (TIB), total sleep time (TST), sleep efficiency (SE), and time awake after sleep onset (WASO); (b) the slope of the correlation lines between the parameters estimated by the two procedures, very close to 1, which demonstrates the linearity of the predictions; (c) the low bias value in the estimates obtained through ACM. Sleep in PD is associated with lower distal skin temperature, efficiency and overall sleep time; greater WASO, activity during sleep and duration of naps and a worse circadian function index. In summary, the ACM device has proven to be clinically useful to evaluate sleep in an objective manner, thanks to the integrated management of different complementary variables, having advantages over conventional actigraphy.

## Introduction

Sleep disorders constitute one of the most common non-motor symptoms of Parkinson's Disease (PD), with a prevalence of up to 90% (1), affecting the quality of life of patients to a large extent (2). The development of devices and procedures for sleep monitoring in this population is essential in order to implement treatment strategies aimed at improving the quality of life in patients with PD.

Traditionally, sleep detection has been carried out using polysomnography (PSG), a technique considered to be the *gold standard* for evaluating the sleep disorders. However, this technique presents certain disadvantages: its high cost, short recording time (at most, one or two nights, with no recordings during the day), the inconvenience of the sensor wiring and the fact that it forces the patient to sleep under unusual conditions and in a strange environment, which may condition the first night effect, making it difficult to fall asleep (3). For these reasons, the search for alternative techniques to PSG represents an urgent need. Among these alternatives, actigraphy, which uses an accelerometer to detect movement and a series of algorithms to infer sleep, has been the most successful technique in recent years. This technique permits the objective, non-invasive recording of the sleep-wake pattern over long periods of time and estimating parameters such as total sleep time (TST), sleep efficiency (SE) and sleep latency (SL), with good agreement with PSG (4, 5). However, sleep detection via algorithms based solely on movement shows important weaknesses. In general, actigraphy has been demonstrated to be very sensitive in detecting sleep, but its capacity to detect wake states during the night (specificity) is not as high (6), as it tends to overestimate sleep by considering moments of immobility in bed, or even apparent immobility due to the removal of the sensor, as sleep (5, 7). That is the reason why it loses clinical importance when studying specific pathologies, such as insomnia, because the subject may remain awake for long periods of time without barely moving (7).

In addition, the main sleep parameters derived from the different actigraph models are inferred based on measurements processed using different procedures (8), such as the Zero Crossing Mode (ZCM), Time Above Threshold (TAT) and Proportional Integral Mode (PIM), which are calculated with different frequencies, sometimes (but not always) using filters, depending on the device, and applying algorithms based on different procedures, such as Cole-Kripke, Sadeh or UCSD (8–10). These algorithms often tend to be specific to different devices, age groups and sleep pathologies, which makes it difficult to compare results, requires prior knowledge of the pathology being evaluated and complicates conducting studies with large population groups using the same technique.

To make up for the lack of precision associated with the recording of a single variable, multivariable recordings under ambulatory conditions have recently been suggested (11). These procedures, generically referred to as Ambulatory Circadian Monitoring (ACM), are based on the integration of a combination of variables, such as skin temperature, motor activity and body position (12). The peripheral temperature rhythm measured on the wrist is especially important in this procedure, since it is not only very useful for detecting sensor removal, it is also a good sleep marker (11, 13), having been used to evaluate circadian rhythms in several physiological and pathological conditions, such as metabolic syndrome (14), obesity (15), and sleep apnea (16).

The development of wearable devices has increased interest in the use of ambulatory methods to detect sleep disorders in PD more objectively than those permitted by scales evaluating sleep quality (17, 18). In addition, these sensors can be used to monitor and detect early symptoms of PD, which will facilitate the intensive long-term monitoring at home, offering the possibility of providing individualized medical care and, at the same time, making it an effective, affordable procedure to monitor the progression of PD.

The main objective of this work is to validate an ACM device for the detection of sleep and wake states and apply it to the evaluation of sleep quality in patients with PD. To do this, a polysomnographic validation study was conducted in a preliminary phase on a large group of patients of different ages, genders and sleep pathologies, followed by a pilot study to apply this methodology to patients with Parkinson's disease.

## Methods

### Study Populations

Two separate studies have been carried out, involving different subjects: one for the polisomnographic validation for sleep detection of the ACM device in a Sleep Unit and a second one for sleep-wake rhythm assessment in patients with PD under normal-living conditions.

For Kronowise polisomnographic validation, seventy patients were studied (26 women, 44 men, with an age range of 11–86 years), recruited among those patients who attended the Sleep Unit at Ribera de Alzira University Hospital (Spain), by strict order of reception, between February and March 2016. Ambient room temperature was kept at 23 ± 1°C, and the same experimental protocol was applied to all subjects. PSG was conducted on these patients to attempt to diagnose the cause of their sleep problems. From all monitored subjects, 9 showed no sleep pathology after the PSG. The remaining subjects were diagnosed as follows: obstructive sleep apnea (38), periodic leg movements (PLM) (5), onset and maintenance insomnia (5) and apnea+PLM (13). Their characteristics are described in Table 1.

**Table 1:** Characteristics of the subjects participating in the polysomnographic validation study.

	**Mean ± SEM**	**Range**
Age (years)	55.67 ± 1.85	11–86
Gender (F/M)	26/44	
BMI	29.29 ± 0.66	19–47
No pathology	9	
Severe apnea	23	
Moderate apnea	8	
Mild apnea	7	
PLM	5	
Insomnia	5	
Apnea + PLM	13	

*Mean values ± standard error of the mean (SEM). PLM, periodic leg movement*.

For the sleep-wake rhythm assessment in patients with PD under ambulatory conditions, a cross-sectional design was used including 30 adult volunteers: 15 patients with PD, who met the diagnostic criteria according to the MDS 2015 criteria (PD group) (19) and 15 healthy control subjects who matched the same demographic characteristics (control group). The patients with PD were selected by convenience sampling among those who attended the Movement Disorder Unit at Virgen de las Nieves University Hospital in Granada (HUVN). Control subjects were recruited among relatives of the University of Murcia's students whose anthropometric and demographic characteristics matched those of the PD subjects. Both groups were instructed to maintain their normal lifestyle during the week of the study. All participants received adequate information on the study and signed an informed consent form before being included. The study was approved by the Ethics Committee at the University of Murcia and HUVN.

All patients were in treatment with L-dopa and/or dopamine agonists. Exclusion criteria were: diagnosis of dementia or severe psychiatric comorbidity, fever or infection over the last 2 weeks, habitual tobacco use or alcohol abuse, diagnosis of diabetes mellitus during ≥10 years or subjected to treatment with insulin for ≥5 years, clinical polyneuropathy, endocrinopathies (thyroidopathies or diseases of the adrenal glands), arterial diseases (Raynaud, thoracic outlet syndrome), treatment with medications for excessive daytime sleepiness (i.e., modafinil), treatment with adrenergic agonists/blockers or a connective tissue disease that could affect skin temperature. None of them were shift workers or had crossed several time zones during the month prior to testing. The same exclusion criteria applied to the control group. Trained interviewers evaluated the severity of PD according to the Hoehn and Yahr scale (20). The clinical disability of the patients was evaluated according to the Unified Parkinson's Disease Disease Scale Rating (UPDRS) and its corresponding subscales. The patients with PD also completed non-motor and sleep evaluations, using the second version of the Parkinson's Disease Sleep Scale (PDSS-2) and the Parkinson's Disease Quality of Life Questionnaire (PDQ-39). Subjects in both groups completed the Pittsburgh Sleep Quality Index (PSQI) and the Epworth Sleepiness Scale (EES). The equivalent dose of Levodopa (LED) was determined in patients with PD using standardized protocols (21). The characteristics of the patients who participated in this study are described in Table 2.

**Table 2:** Characteristics of the patients who participated in the sleep rhythm evaluation study in Parkinson's disease.

	**PD**	**Controls**	***P***
	**Mean ± SEM**	**Mean ± SEM**	
Age (years)	65.53 ± 2.19	60.71 ± 1.97	0.10
Gender (M/F)	12/3	12/3	1.00
Weight (kg)	77.64 ± 2.09	79.43 ± 3.03	0.56
Height (cm)	170.43 ± 1.78	175.29 ± 2.34	0.11
BMI	26.71 ± 0.56	25.77 ± 0.69	0.37
PD progression (years)	11.27 ± 1.49		

*Mean values ± standard error of the means (SEM). The differences between means were evaluated using a Student's t-test, with values being considered significant at p < 0.05. BMI, body mass index*.

### Polysomnographic Validation of the ACM Device for Sleep Detection

A conventional PSG test was administered in the sleep unit as the standard of reference to evaluate the reliability of the ACM device for sleep detection. Electroencephalographic (EEG) activity was recorded by surface electrodes placed on the scalp in the central, frontal and occipital regions by means of monopolar leads with a reference electrode located on the contralateral mastoid process (F4-M1, F3-M2, C4-M1, C3-M2, O2-M1, O1-M2), based on the international 10–20 system, according to the recommendations of the American Academy of Sleep Medicine (AASM Scoring Manual 2015). Electromyographic (EMG) activity was recorded by electrodes in the submental region and on both anterior tibial muscles. A modified DII lead was used to record the electrocardiogram, whereas eye movements were recorded by two electrodes placed on the left (LOC-M2) and the right (ROC-M2) eye. Respiratory signals included nasal pressure cannulas and nasal and oral thermal sensors to assess nasobuccal flow. Respiratory effort was assessed by means of inductance plethysmography bands on the thorax and abdomen, including the signal of the sum of both. The snoring signal was obtained by filtering the nasal cannula signal (PTAF, *Protech*). Blood oxygen saturation (SaO2) was recorded by pulse oximetry. PSG data were acquired simultaneously from 20 different channels at 30 s per page, for ~8 h, using a 44-channel *Grass*®*Comet-PLUS*® *EEG/PSG* equipment (Natus Medical Incorporated, San Carlos, CA, USA).

Sleep (N1, N2, N3 and REM) and wake stages were classified according to the criteria of the American Academy of Sleep Medicine (AASM *Scoring Manual* 2015). An infrared video camera recorded the entire PSG procedure in a synchronized manner. To synchronize the results of the PSG (one page every 30 s) with the recordings from the ACM KW device (one data recording every 30 s), the infrared sensor of the KW device was used to detect the turning on and off of the video recording.

### Use of an Ambulatory Circadian Monitoring (ACM) Device for Detecting the Sleep-Wake Rhythm

Both for sleep detection during the polysomnographic validation study and to evaluate the sleep-wake rhythm in patients with PD under normal living conditions, an ACM device the size of a wrist watch was used (*Kronowise 3.0*, Kronohealth, S.L., Spain). In the case of patients with PD, it was placed on the less affected hand, and in the case of the control subjects and patients in the PSG validation study, it was worn on the non-dominant hand. The main variables recorded by this device were wrist skin temperature, triaxial acceleration, wrist position, exposure to light on three spectral bands (visible, blue light with a wavelength of 460–490 nm and infrared light >800 nm). An electronic record (event marker) was kept that could be used by the subjects as an electronic diary. The sampling frequencies were: 10 Hz for the acceleration and time in movement measurements, 1 Hz for skin temperature and exposure to light and 0.033 Hz (1 reading per time period) for wrist position and event marker. The data captured over the course of a week (~23,000,000 data points) were internally processed and saved in 30-s time periods. The technical characteristics of the ACM Kronowise 3.0 device have been previously described by Madrid-Navarro et al. (18). Briefly, the device is equipped with:

1. A temperature sensor with an accuracy of ±0.1°C at 25°C and a resolution of 0.0635°C.
2. A MEMS calibrated triaxial accelerometer with a linear sensitivity equal to (0.001) g along all three axes and a range of ±2 g. The Y axis of the device was aligned with the radius; the X corresponded to the radial-cubital axis and the Z axis with the palmar-dorsal axis. Based on the information provided by the accelerometer, a total of five groups of motor variables were recorded: (a) tilt of the X, Y, and Z axes, as well as the angle between each axis and the horizontal plane, expressed in degrees; (b) the sum of the degrees of change between the position of the current axis and the previous one; (c) the area under the curve, which proportionally integrates the acceleration values per time period (*proportional integrated mode*, PIM); this variable indicates speed of movement and force, but not the duration or frequency of the movement; (d) time in movement, as the cumulative time above the threshold of 0.05 g (*time above threshold, TAT*) in which a movement on any of the three axes was detected. This parameter ranges from 0 (no movement in any of the three axes, none of the 300 times in which acceleration is sampled in a period of 30 s) to 300 (by adding each movement >0.05 g every time the acceleration is sampled in 30 s, that implies 300 times, scores as 1); (e) the area under the curve for the individual X, Y, and Z acceleration, in order to discriminate among the types of motor activity (i.e., walking, running, writing, etc.).
3. Three light sensors on the front to record visible, infrared and blue light, with a range of between 0.01 and 43,000 lux, with internal self-adjustment according to the level of luminance and suppression of flicker at 50/60 Hz. The ratio between infrared/visible light made it possible to determine the source of light (i.e., natural, fluorescent, infrared, incandescent, or LED light). The sensor for blue light was equipped with a filter that only let circadian light through, which is the one primarily detected by the melanopsin cells of the retina.

Communication with the ACM device was established using *Kronoware 10.0* software (Kronohealth, S.L., Spain) via a USB port. This software allows visual inspection before analysis to eliminate any artifacts and the calculation of circadian and basic sleep parameters. In this study, four calibrated Kronowise devices were used with minimal differences in variables recorded among them (coefficient of variation <4%). The data were converted into a text file for later analysis using the chronobiological software *Circadianware*, implemented on the *Kronowizard* cloud platform (https://kronowizard.um.es/, University of Murcia).

### Automatic Detection of Sleep and Wake States

**Figure 1:**
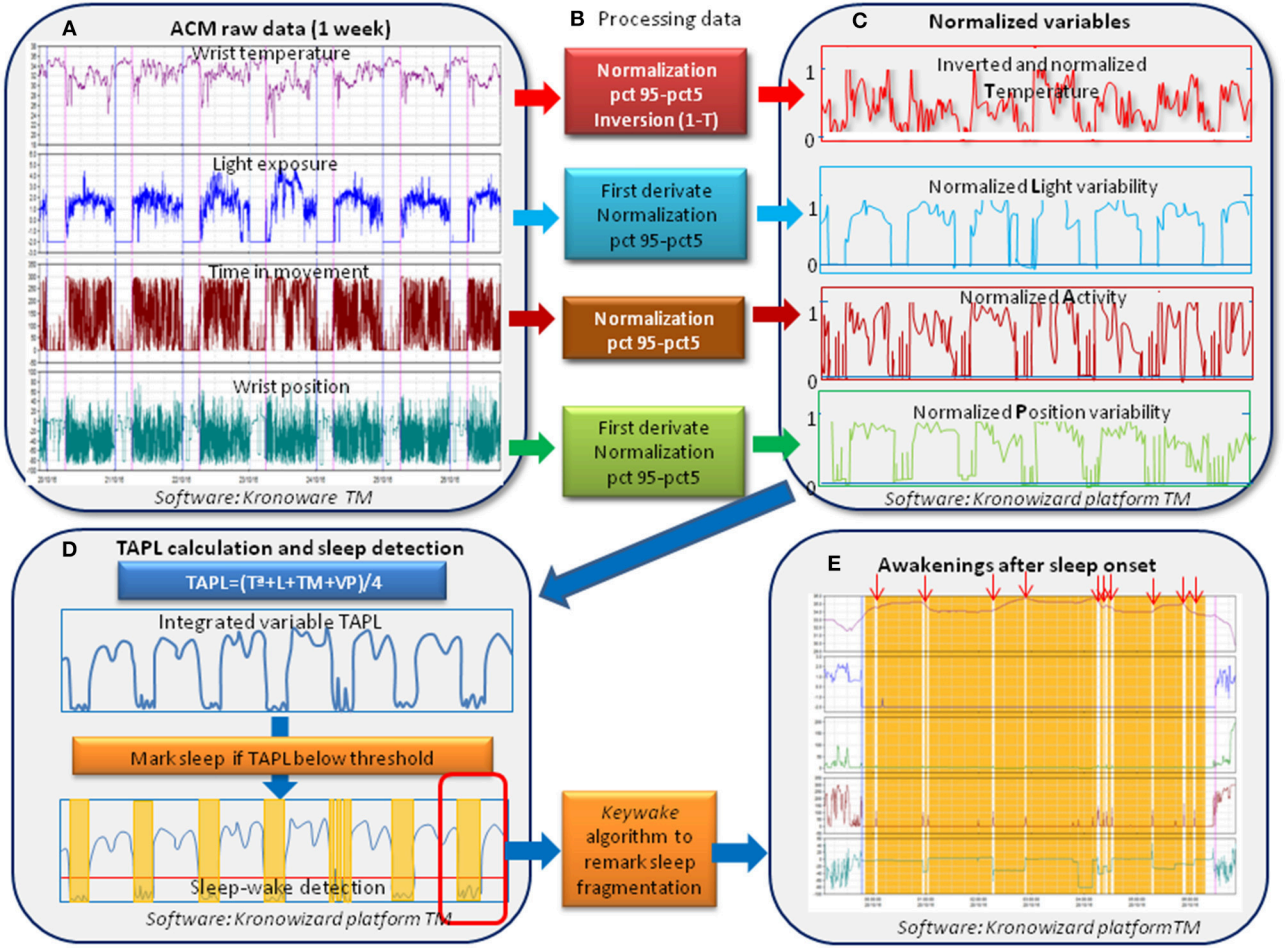
Sequence for data processing and algorithms used for automatic detection of sleep and wake periods. Downloaded raw data from ACM device **(A)** were submitted to a two-phase procedure. The first procedure for automatic sleep and wake **(B,C)** detection used the TAPL algorithm **(D)** (integrating wrist temperature, motor activity, variability in wrist position and variability in light exposure), implemented on the Kronowizard website (https://kronowizard.um.es/, University of Murcia). Given that, WT rhythm is the inverse of the A, P, and L, WT values were inverted before calculating the mean of the four standardized variables. Therefore, a TAPL value of 0 indicates deep rest, characterized by immobility, vasodilation of the skin vessels, and low variability of L exposure, while 1 corresponds to wake, movement, vasoconstriction, and high light variability. A concrete period was classified as sleep when TAPL values fell beneath a preset threshold **(D)**, previously validated by PSG (11). Next, we proceeded to remark sleep episodes using the Keywake algorithm implemented on the Kronowizard website (https://kronowizard.um.es/, University of Murcia) in order to improve the precision of the estimates **(E)**.

To automatically detect sleep and wake periods, a two-phase procedure was used (Figure 1). The first was aimed at the automatic detection of sleep and wake periods using the new TAPL algorithm, a modification of the TAP algorithm (12) to additionally integrate exposure to visible light, implemented on the Kronowizard website (https://kronowizard.um.es/, University of Murcia). As described by Ortiz-Tudela et al. (12), the TAP algorithm is based on the intra-subject standardization of three signals: wrist skin temperature (WT), time of movement (TM), and variability of the position of the X axis (PX) by time period, using the 95th and 5th percentiles as the upper and lower intervals for standardization, respectively. In order to include variability of exposure to visible light (L) in the new TAPL algorithm, the same procedure was used as for its standardization. Given that the WT rhythm is the inverse of the TM, PX, and L, WT values were inverted before proceeding to calculate the mathematical mean of the four standardized variables. Therefore, a TAPL value of 0 was an indicator of deep rest, characterized by immobility, vasodilation of the skin and low variability of L exposure (sleep), while 1 corresponded to a wake state, light and movement (wake). A time period was classified as sleep when the TAPL value fell beneath a preset threshold, previously validated by PSG (11). Once the main sleep and wake periods have been detected by means of TAPL, we proceeded to mark the sleep episodes in order to improve the precision of the estimates, using PSG as the standard. The *Keywake*® algorithm was used to mark these periods, using artificial intelligence and based on the time in movement from the 4 min before and 2 min after each time period evaluated. All these calculations are implemented on the *Kronowizard* platform (https://kronowizard.um.es/, University of Murcia).

### Calculation of Circadian Parameters

To characterize the circadian pattern of PD patients and the control group, a non-parametric analysis was carried out as described in the literature (22), which includes the following parameters:

- Interdaily stability (IS) throughout the recording period calculated as follows:
  $$\begin{array}{l} \text{IS}=\frac{\text{n}\sum_{\text{h}=1}^{\text{n}}{\left({\bar{\text{x}}}_{\text{h}}-\bar{\text{x}}\right)}^{2}}{\text{p}\sum_{\text{i}=1}^{\text{n}}{\left({\text{x}}_{\text{i}}-\bar{\text{x}}\right)}^{2}} \end{array}$$
  Where n is the total number of data, p the number of data per day, ${\bar{x}}_{h}$ the mean value of this time point, $\bar{x}$ the mean value of the complete series of data, and *x*_*i*_ the individual data. This index varies from 0 for Gaussian noise and 1 for perfect stability, where the rhythm repeats itself exactly, day after day.
- Intradaily variability (IV), indicates the fragmentation of the rhythm and was calculated according to the following formula:
  $$\begin{array}{l} \text{IV}=\frac{\text{n}\sum_{\text{i}=2}^{\text{n}}{\left({\text{x}}_{\text{i}}-{\text{x}}_{\text{i}-1}\right)}^{2}}{\left(\text{n}-1\right)\sum_{\text{i}=1}^{\text{n}}{\left({\text{x}}_{\text{i}}-\bar{\text{x}}\right)}^{2}} \end{array}$$
  Where n represents the total number of data, *x* the mean value of the complete series of data, *x*_*i*_ the individual data, and *x*_*i*−1_ the data immediately before to *x*_*i*_. IV values oscillate between 0 (when the variable is not fragmented) and 2 (Gaussian noise).
- The mean value and the central hour of the 10 consecutive hours with the lowest values (L10V and L10T, respectively) of WT and sleep probability (probability that a subject is asleep at a given time), and the mean value and central hour of the 10 consecutive hours with the highest values (M10V and M10T, respectively) of the acceleration calculated as PIM, TM, and light exposure (LE). All these indexes are indicators or the level of activation during the wake period.
- The mean value and the central hour of the 5 consecutive hours with the lowest values (L5V and L5T, respectively) of acceleration, TM and LE, and the mean value and central time of the 5 consecutive hours with the highest values (M5V and M5T, respectively) of WT and sleep probability. All these indexes are markers of stillness and depth of sleep.
- The standardized relative amplitude (RA) has been calculated as the difference between VM10 and VL5, divided by the difference between the two extreme percentiles, Pc95th M10V-Pc5th L5V for acceleration, TM and LE. The percentiles were obtained from a population of 90 healthy adults previously recorded using the KW3 device (https://kronowizard.um.es/, University of Murcia). The reference values for acceleration were: 40 and 1 g for the 95th and 5th percentiles, respectively; 200 and 2 counts/30 s for the time in movement; and 3 (log lux) and 0 (log lux) for light exposure. The reference values were rounded off to the nearest upper and lower whole values for the Pc95th and Pc5th, respectively. Given that the skin temperature and the sleep probability exhibit an inverse pattern to motor activity and light exposure, their RA referred to the difference between M5V and L10V, considering the 95th percentile for M5V and the 5th percentile for L10V (M5V-L10V)/(Pc95th M5V-Pc5th L10V). In this case, the reference values for skin temperature were 35–30°C and 1 and 0 for the sleep probability, according to the 95th and 5th percentiles, respectively. As an integrated index to differentiate between PD and C, the quotient between M10V of acceleration/L5V of time in movement (A/T) was used, as previously described (18).

In order to obtain a global index of the robustness of the circadian system (inverse to chronodisruption), the circadian function index (CFI) (12) was calculated as the mean of IS, IV and RA, but the IV values were previously inverted and standardized between 0 and 1. Therefore, a CFI of close to 1 indicates a high amplitude rhythm, that is unfragmented and stable.

### Calculation of Sleep Parameters

#### Detection of the Sleep Period

**Figure 2:**
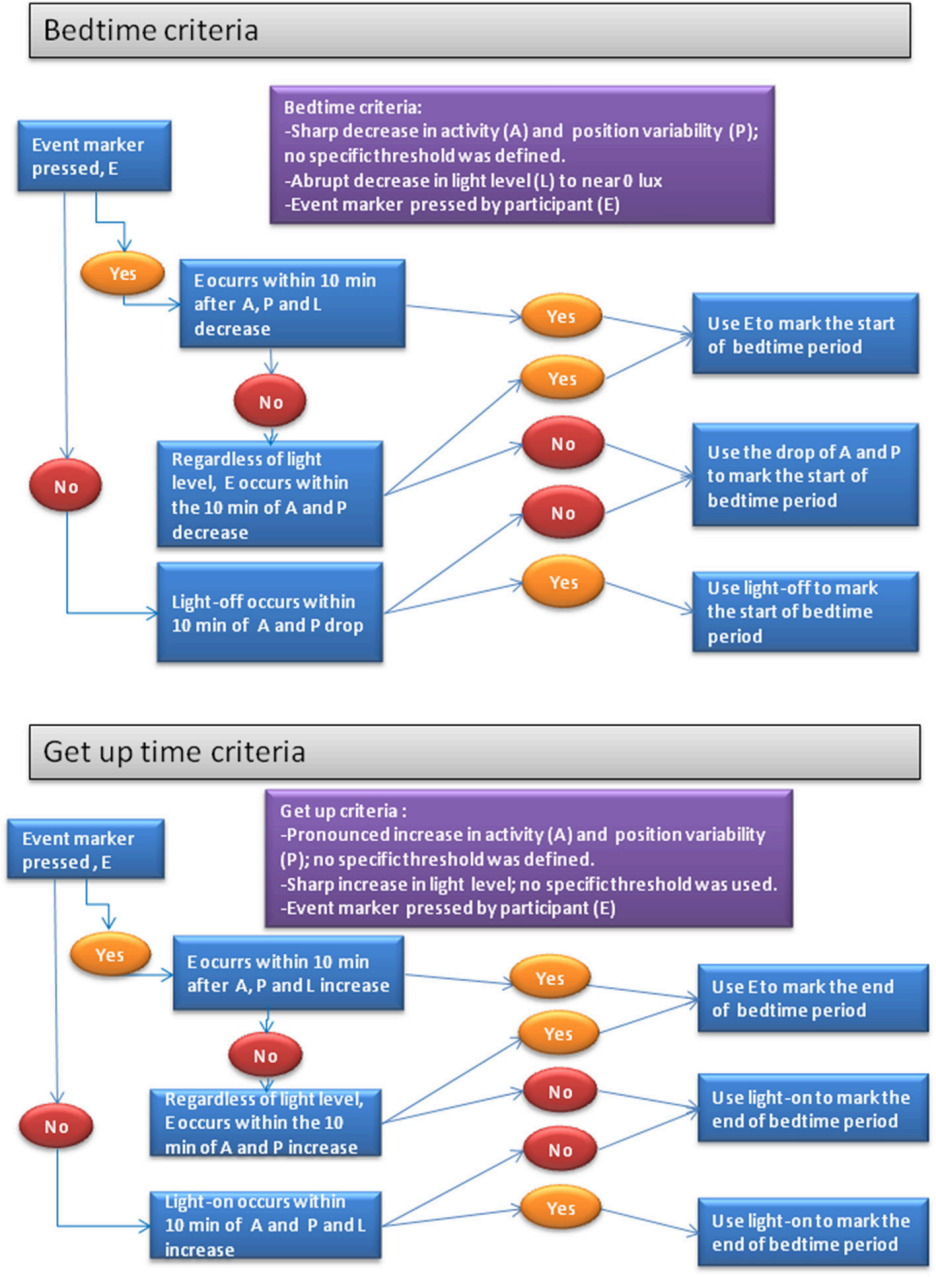
Decision trees for the marking, using criteria objectifiable by an expert, of the sleep interval defined as the time between the voluntary start of sleep (bed time) and the end of the time spent in bed (get up time). The expert uses the criteria of: the event marker (E), lights on and off (L), motor activity (A), and stability of wrist position (P).

While the sleep and wake periods were automatically detected as previously described, the precise moment at which the subject went to bed (bed time, BT) and got up (get up time, GUT) was determined differently in the laboratory PSG validation study than in the ambulatory study on patients with PD. In the first case, the start and end of the time period in bed was inferred automatically, using the sharp increase in the ratio between infrared/visible light generated by turning off the visible light and turning on the infrared camera during the PSG validation period. However, in the laboratory study and given the great variability of habits related to going to bed and getting up in patients with PD, their period of time in bed was recorded manually, according to the following procedure, which is described in Figure 2. Bed time was defined using the following information: drop in activity level, visible light off and, if appropriate, the event marker, as shown in the top of Figure 2. Get up time was defined using the following indicators: increase in activity level, decrease in skin temperature, increase in light level above 1.0 μW/cm^2^ and, if applicable, an event marker (bottom part of Figure 2). The rest of the sleep parameters were calculated automatically, as described below:

- Time in bed (TIB): period of time between BT and GUT.
- Sleep onset (SO): first time period marked as sleep after BT.
- Awake time (AT): the last time period marked as sleep before GUT.
- Sleep interval (SI): time between SO and AT.
- Sleep onset latency (SOL): total time in minutes between BT and SO.
- Wake after sleep onset time (WASO): total minutes marked as awake after SO.
- Total sleep time (TST): time marked as sleep during the sleep interval.
- Sleep efficiency (SE): percentage of time asleep with regard to the time in bed; SE = (TST/TIB)^*^100.
- Number of awakenings: number of awakenings equal to or longer than 30 s per hour of sleep interval.
- Total time in movement (TTM): total minutes in the sleep interval during which movement has been detected.
- Time in movement index (TMI): average per time period in seconds in which movement has been detected (expressed in s/30 s).
- Sleep acceleration index (SAI): mean total acceleration time per time period (expressed in g/30 s).
- Wrist sleep temperature (WST): mean skin temperature during the sleep interval.
- Napping time (NT): sleep time outside the main sleep period.
- Napping frequency (NF): number of nap episodes per day.

### Statistical Analysis

The statistical comparisons between the sleep parameters detected by PSG and ACM were carried out using different methods, according to the objective of the analysis: (1) The mean values of the sleep parameters obtained by ACM were compared to those of PSG, using a paired samples Student's *t*-test; (2) Associations between values calculated by PSG and ACM were analyzed using Pearson's correlation analysis; (3) To show the degree of concordance between the sleep parameters determined by PSG and ACM, a Bland-Altman analysis was used, representing the difference between the PSG-ACM estimates on the Y axis and the mean of both values on the X axis, along with the 95% confidence interval and the deviation from the mean of the estimates; (4) The comparison between the sleep parameters in patients with PD and the control subjects was carried out by means of a Student's *t*-test for non-paired samples. The data were processed and graphically represented using Microsoft Office Excel 2016, and were statistically analyzed using SPSS v20.0 software (SPSS, Inc. Chicago, IL, USA).

## Results

### Characteristics of the Subjects Included in the Study

The main characteristics of the subjects who participated in the PSG validation study are described in Table 1. In the PSG-monitored patients, the duration was at least 420 min. In most cases, the clinical suspicion was confirmed, diagnosing obstructive sleep apnea in a total of 38 patients, PLM in 5, insomnia in 5 and PLM + obstructive sleep apnea in 13.

The characteristics of the patients included in the sleep rhythm evaluation study in PD are shown in Table 2, with ages between 44 and 78 years and no significant differences in age or gender as compared to the control group. The mean duration of the disease in the group with PD was 12 ± 1.8 years (with a range of 3–20 years). None of the participants had previously been diagnosed with RLS or PLM and one patient had mild obstructive sleep apnea.

### Polysomnographic Validation of Sleep Detection by ACM

**Figure 3:**
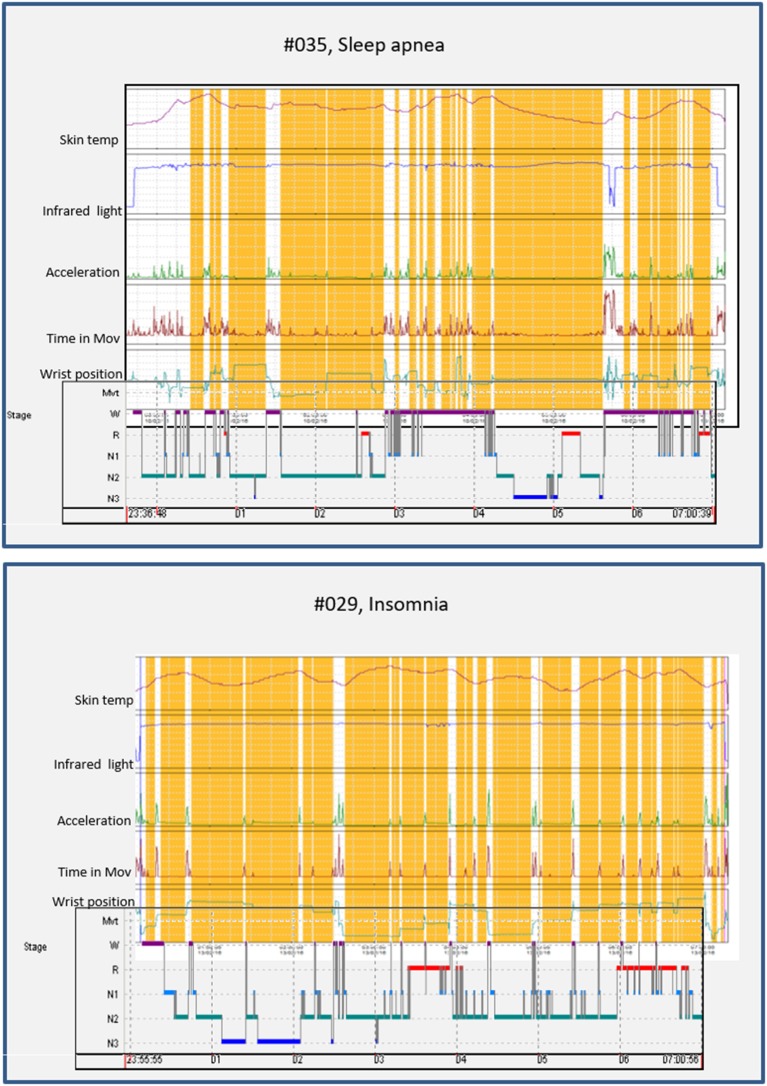
Representative examples of two sleep pathologies (apnea and insomnia), for which a comparison between the hypnogram determined by PSG and the sleep pattern obtained by ACM recording is shown. The sleep detected by the ACM device is shown in orange, while the awakenings appear in white. The estimation of sleep and wake episodes were determined automatically based on the integration of sleep temperature, light exposure (visible and infrared), time in movement, and hand position. The corresponding hypnogram has been superimposed on the bottom of each panel to facilitate comparison.

Figure 3 shows the representative hypnograms for two patients with different sleep pathologies (apnea and insomnia), simultaneously recoded by ACM and PSG. The start and end of the PSG recording can be observed in the ACM panel by the sharp increase in the intensity of infrared light. A high level of concordance is observed between the awakenings detected by ACM and those shown on the hypnogram obtained through PSG. The sleep periods and awakenings were automatically detected using the *Circadianware* software based on the TAPL algorithm. The fine rescoring of awakening during sleep periods was performed by means of the *Keywake*® algorithm implemented on the *Kronowizard* website (https://kronowizard.um.es/, University of Murcia).

**Figure 4:**
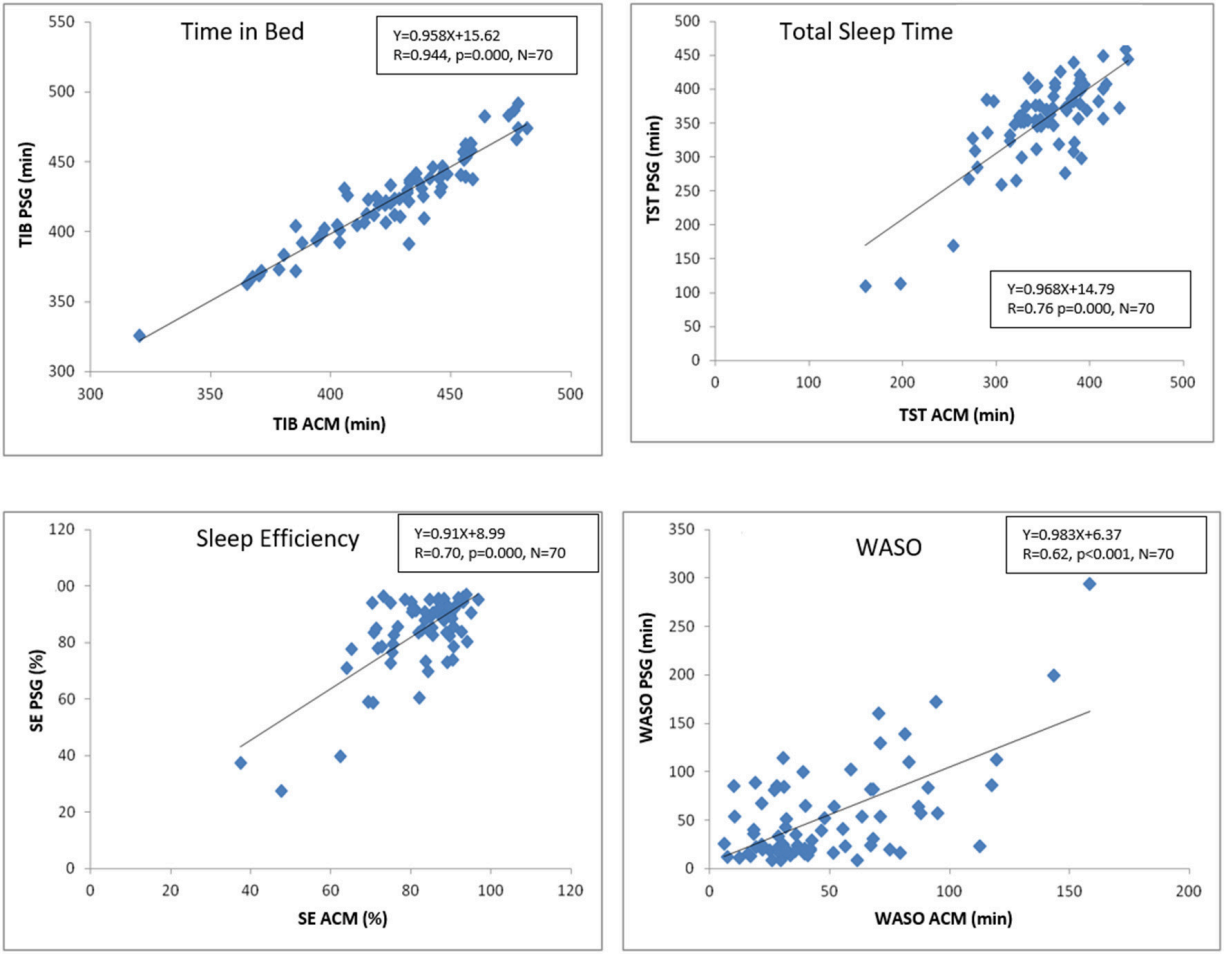
Pearson correlations showing the correspondence between the main sleep parameters estimated by ACM and PSG. TIB, time in bed; TST, total sleep time; SE, sleep efficiency; WASO, wake time after sleep onset. The graph indicates the corresponding equation, its R value and its probability.

For the comparative analysis between PSG and ACM, to begin with, the same parameters were selected, estimated by each of the two techniques, with no statistically significant differences detected between the two procedures in terms of time in bed (TIB, 430.4 ± 3.8 min KW vs. 424.5 ± 4.01 min PSG, *p* = 0.27), total sleep time (TST, 351.9 ± 5.2 min KW vs. 352 ± 4.7 min PSG, *p* = 0.99), sleep efficiency (SE, 0.82 ± 1.0 KW vs. 0.83 ± 0.1 PSG, *p* = 0.59), and time awake after sleep onset (WASO, 50.0 ± 3.8 min KW vs. 56.6 ± 3.5 min PSG, *p* = 0.34). As shown in Figure 4, there was a strong significant positive correlation between TIB estimated by ACM and that detected by PSG. Likewise, a statistically significant positive correlation was found between TST, SE, and WASO.

**Figure 5:**
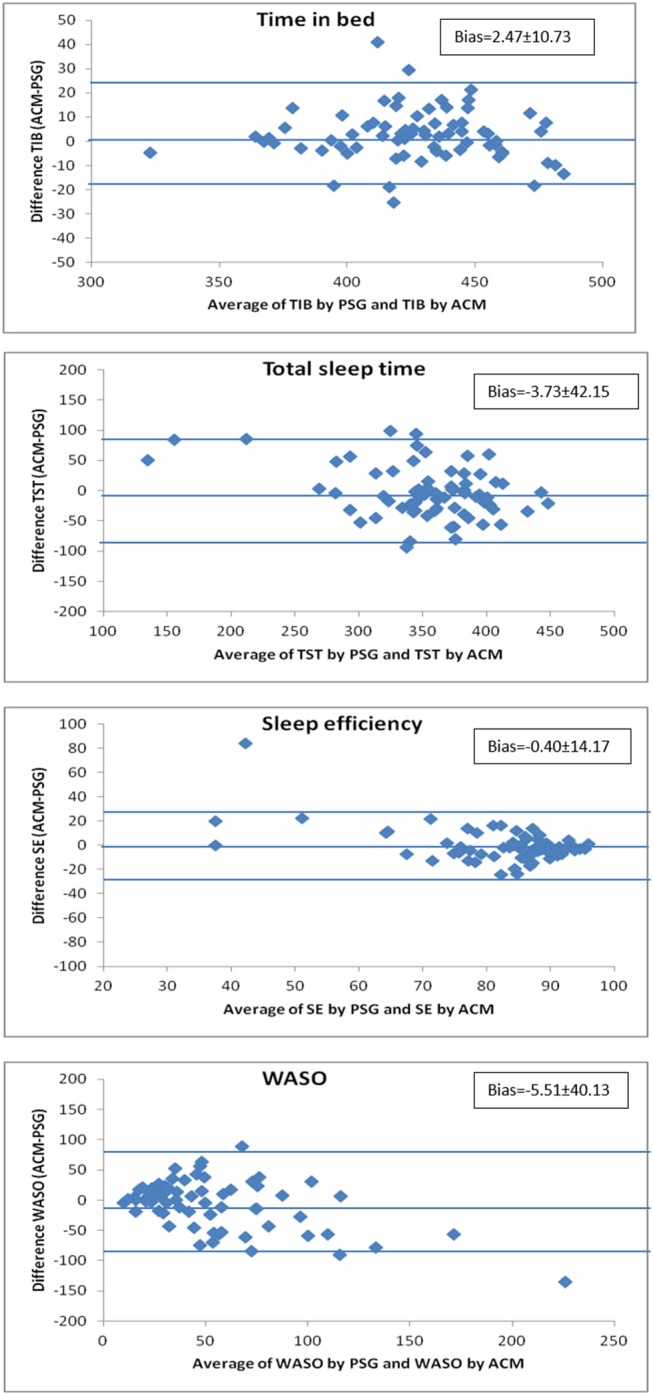
Bland-Altman representation, comparing the deviations in the estimates generated by ACM and PSG. ACM overestimates time in bed by 0.58% and underestimates total sleep time by 1.05%, sleep efficiency by 0.48%, and WASO (wake time after sleep onset) by 10.5%. Each of the graphs shows with horizontal lines the mean deviation ± 1.96 SD.

The Bland-Altman analysis (Figure 5) shows that, on average, ACM overestimates TIB by 2.47 min, which represents 0.58% of the mean TIB value. In the case of TST detection, the underestimation with ACM is 3.73 min (1% of the mean), while for SE, barely 0.48% is underestimated. Finally, the Bland-Altman analysis for WASO shows an underestimation of this parameter of 5.51 min, which represents 10.5% of its mean value. It also provides information on individual agreement between ACM and PSG. Sixty nine subjects (98.6%) presented differences lower than 43 min (10% of TIB mean) for TIB; lower than 35 min for TST (10% of the mean) in 48 individuals (69%); 49 individuals (70%) exhibited differences lower than 8% for SE (10% of the mean). Finally, in the case of WASO, differences lower than 25 min (50% of WASO) were found in 35 individuals (50%).

In order to determine whether the value obtained directly from the ACM device for the time in movement index (TMI, obtained from the TAT mode) could be used as a reliable predictor of the sleep parameters recorded by PSG, and thus prevent any implicit errors or bias in their indirect estimates by ACM, a correlation analysis was carried out between the TMI during sleep and the parameters indicating sleep quality calculated by PSG. While the correlations between TMI and the parameters estimated by PSG are statistically significant, their level of significance does not reach the values obtained when comparing the same sleep parameters calculated by ACM and PSG (see the Pearson's correlations on the previous page). Accordingly, TMI correlates positively with WASO (*r* = 0.51, *p* < 0.001) and negatively with sleep efficiency (*r* = −0.51, *p* < 0.001), both parameters calculated by PSG. TMI also demonstrated a statistically significant negative correlation with REM sleep time (*r* = −0.48, *p* < 0.001) and time in the N3 phase (*r* = −0.37, *p* < 0.01).

### Sleep-Wake Rhythm in Patients With Parkinson's Disease

**Figure 6:**
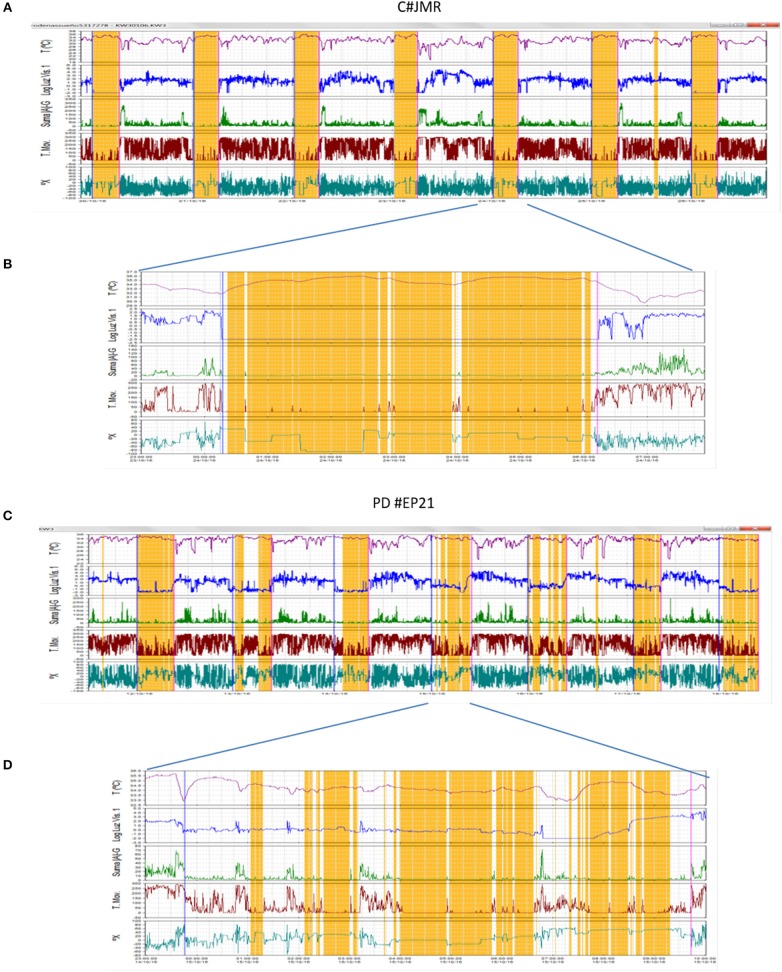
Weekly recording **(A,C)** and one-night recording **(B,D)** via ACM of wrist temperature (red line), exposure to visible light (blue line), acceleration (green line), time in movement (brown line), wrist position (dark green line), and estimated sleep (orange bars), representative of two subjects monitored in the study: one control subject **(A,B)** and a patient with PD **(C,D)**. Surprising is the great fragmentation of sleep, accompanied by high levels of movement, frequent lights-on episodes and temperature drops of the skin during sleep that were observed in the patient with PD.

The ACM KW device allows the non-invasive recording of fifteen rhythmic variables, as described by Madrid-Navarro et al. (18). Among them, five complementary variables were selected to characterize the PD and C sleep pattern: wrist skin temperature (WT), movement acceleration, wrist position, TM and exposure to visible light (environmental synchronization). The integration of the information in the TAPL algorithm made it possible to infer sleep and wake states, as previously described. Figure 6 shows two representative recordings (a full week and one representative night) for the PD and C groups. In general, the PD patients showed great sleep fragmentation accompanied by a high level of movement and frequent lights-on episodes, and alterations in thermoregulation were also detected in a high percentage of them.

**Figure 7:**
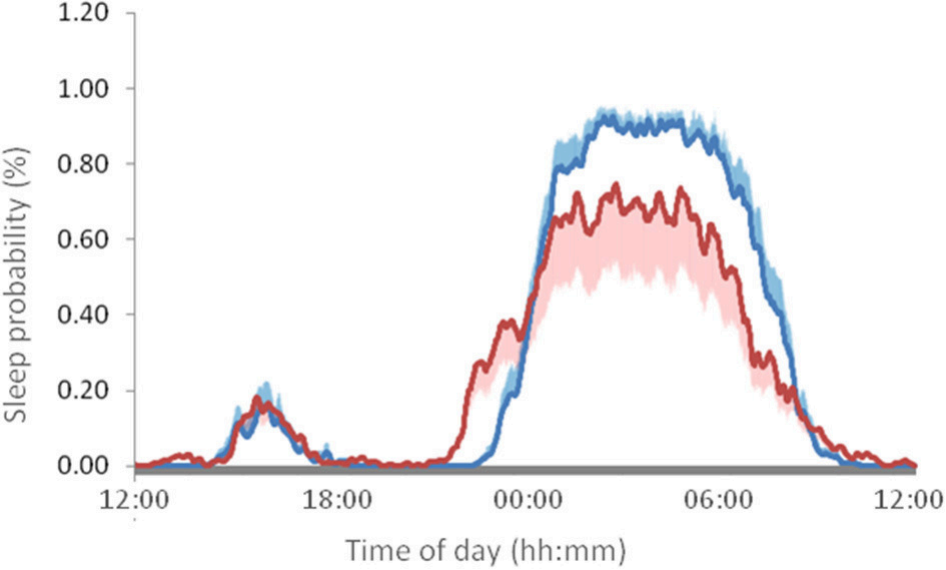
Mean 24-h wave of sleep probability in patients with PD (red line) and healthy control subjects (blue line). The values represent the mean ± SEM for 15 subjects in each condition, monitored every 30 s for 7 full days.

The circadian sleep-wake pattern shown in Figure 7 and Table 3 is significantly different between the patients and the control subjects. A slight phase advance in sleep onset and offset and a lower probability of sleep during the night were observed. Subjects with PD showed less regularity, a lower probability of nighttime sleep (M5V), less contrast between day and night (RA) and greater chronodisruption, as demonstrated by lower CFI values (Table 3).

**Table 3:** Circadian parameters of the probability of sleep, motor acceleration, and time in movement variables.

**Circadian parameters**	**Sleep**			**Acceleration**			**Time in movement**		
	**PD**	**C**	**P**	**PD**	**C**	**P**	**PD**	**C**	**P**
Mean ± SEM	**0.23 ± 0.02**	**0.28 ± 0.01**	**0.0047**	**10.10 ± 1.11**	**14.05 ± 0.93**	**0.0086**	10.22 ± 0.80	10.06 ± 0.40	0.8528
M5V	**0.68 ± 0.05**	**0.90 ± 0.01**	**0.0000**	17.99 ± 2.03	26.46 ± 2.55	0.118	17.00 ± 1.15	17.20 ± 0.71	0.8764
M5T	3:58 ± 0:37	3:53 ± 0.14	0.9117	11:04 ± 0:53	13:36 ± 1:02	0.0659	13:02 ± 0:51	13:03 ± 0:51	0.9997
L10V	0.03 ± 0.01	0.02 ± 0.01	0.4934	4.54 ± 0.66	5.25 ± 0.50	0.3812	4.62 ± 0.68	3.23 ± 0.26	0.0585
L10T	14:27 ± 0:43	14:38 ± 0:27	0.8183	4:49 ± 1:28	3:11 ± 0:16	0.2677	4:43 ± 1:20	3:25 ± 0:15	0.3345
M10V	**0.51 ± 0,04**	**0.65 ± 0.02**	**0.0017**	**14.75 ± 1.63**	**21.86 ± 1,49**	**0.0024**	14.67 ± 1.04	15.62 ± 0.59	0.4137
M10T	3:17 ± 0:27	3:27 ± 0:12	0.7542	14:38 ± 0:25	14:29 ± 0:28	0.8166	14:55 ± 0:30	14:32 ± 0:30	0.5984
L5V	0.00 ± 0.00	0.00 ± 0.00	0.3052	3.17 ± 0.51	2.48 ± 0.32	0.2440	**2.75 ± 0.61**	**0.52 ± 0.05**	**0.0008**
L5T	13:09 ± 1:00	15:52 ± 1:03	0.0672	3:48 ± 0:27	3:56 ± 0:15	0.7947	3:45 ± 0:27	3:57 ± 0.15	0.6972
IS	**0.55 ± 0.04**	**0.76 ± 0.02**	**0.0000**	0.33 ± 0.03	0.34 ± 0.02	0.7553	0.42 ± 0.04	0.48 ± 0.02	0.2132
IV	0.11 ± 0.01	0.08 ± 0.01	0.1285	**0.36 ± 0.02**	**0.29 ± 0.03**	**0.0382**	0.28 ± 0.02	0.25 ± 0.01	0.1961
RA	**0.65 ± 0.05**	**0.88 ± 0.01**	**0.0000**	**0.29 ± 0.05**	**0.48 ± 0.04**	**0.0010**	**0.65 ± 0.05**	**0.88 ± 0.01**	**0.0130**
CFI	**0.71 ± 0.03**	**0.87 ± 0.01**	**0.0000**	**0.48 ± 0.02**	**0.56 ± 0.02**	**0.0098**	**0.63 ± 0.03**	**0.70 ± 0.02**	**0.0044**

*The values are expressed as the mean ± SEM. M5V and M5T, mean value and central hour of the 5 consecutive hours with the maximum values; L10V and L10T, mean value and central hour of the 10 consecutive hours with minimum values; M10V and M10T, mean value and central hour of the 10 consecutive hours with the maximum values; L5V and L5T, mean value and central hour of the 5 consecutive hours with the minimum values; IS, interdaily stability; IV, interdaily variability; RA, standardized relative amplitude; CFI, circadian function index; Differences with p < 0.05 on a Student's t-test are shown in bold type*.

The sleep parameters calculated from ACM are shown in Table 4. No significant differences were observed in time in bed, sleep interval, sleep latency or sleep fragmentation (number of awakenings); however, statistically significant differences were seen in all indexes related to sleep efficiency, such as greater WASO, total time in movement and time in movement index, lower temperature during sleep and longer duration and greater frequency of naps.

**Table 4:** Main sleep parameters.

	**PD**	**Controls**	**P**
	**Mean ± SEM**	**Mean ± SEM**	
Time in bed, TIB (min)	457.27 ± 21.96	457.95 ± 13.07	0.9784
Sleep latency, SL (min)	15.95 ± 2.68	10.37 ± 1.97	0.0934
WASO (min)	**110.38 ± 12.43**	**48.55 ± 5.02**	**0.0001**
Total sleep time, TST (min)	**307.03 ± 21.15**	**386.30 ± 11.22**	**0.0019**
Sleep efficiency, SE (%)	**67.23 ± 2.84**	**84.52 ± 1.21**	**0.0000**
Awakenings (No.)	3.36 ± 0.40	3.11 ± 0.25	0.5906
Total time in movement (min)	**29.82 ± 4.84**	**8.46 ± 0.82**	**0.0001**
Time in movement index, TMI (s/30s)	**1.94 ± 0.26**	**0.57 ± 0.05**	**0.0000**
Acceleration index, SAI (g/30 s)	2.83 ± 0.32	2.52 ± 0.31	0.4780
Temperature during sleep (°C)	**33.57 ± 0.21**	**34.15 ± 0.16**	**0.0312**
Visible light during sleep (lux)	3.89 ± 2.37	0.44 ± 0.24	0.1450
Blue light during sleep (lux)	1.52 ± 0.99	0.13 ± 0.08	0.1589
Nap frequency (No./day)	**0.91 ± 0.15**	**0.45 ± 0.09**	**0.0112**
Duration of naps (min)	**33.33 ± 6.19**	**15.81 ± 3.94**	**0.0199**

*Mean values and SEM of the main sleep parameters obtained from 7 days of recording of the sleep-wake rhythm in 15 patients with Parkinson's disease and 15 healthy control subjects (105 nights per experimental condition). Differences with a Student's t-test value of p < 0.05 are shown in bold type*.

## Discussion

This article presents a new method for detecting the sleep-wake rhythm based on the data provided by an ACM (KW) multisensor device validated in comparison to a night PSG. Its usefulness under ambulatory conditions is evaluated for detecting sleep alterations in a neurodegenerative disease, such as PD.

Ambulatory circadian monitoring using a combination of sensors, including thermometry, actimetry, and light exposure, integrated into the TAPL algorithm, is a useful tool for evaluating the main sleep parameters: TIB, TST, SE, and WASO, without the need to resort to different specific algorithms for each sleep pathology and with better predictive capacity than conventional actimeters based solely on the movement of the subject.

The findings presented in our study show the capacity of a multi-channel ACM device to monitor the sleep-wake rhythm in patients with PD while they live their normal lives. Sleep in PD is associated with a lower distal skin temperature, efficiency and overall sleep time; greater WASO, activity during sleep and duration of naps and a worse circadian functioning index.

The ACM device makes possible to estimate the main sleep parameters while the subjects develop their normal life, as demonstrated by: (a) the lack of significant differences between the mean values detected by PSG and ACM for TIB, TST, SE, and WASO; (b) the slope of the regression lines between the parameters estimated by the two procedures are very close to 1, which demonstrates the linearity of the predictions; (c) the low bias value in the estimates obtained through ACM respect.

Actigraphy based on the movement of a part of the body has been widely studied and proposed by the AASM as an appropriate method to evaluate circadian sleep alterations (23). However, unlike ACM techniques, actigraphy presents serious limitations in terms of evaluating subjects with sleep pathologies or neurodegenerative diseases of a non-circadian origin: (a) actigraphy tends to underestimate awakenings in which the subject remains immobile (7); (b) there is no linear relationship between the estimates from actigraphy and PSG; as a result, the more important the sleep alteration is, the greater the bias is between the two readings (24); (c) actigraphy requires the use of specific algorithms for different population and pathology groups (8); (d) the detection of sleep periods can be confused with the sensor having been removed, which requires the patient to keep a sleep diary (8, 25); (e) it provides no information about autonomic alterations or exposure to synchronizers of the circadian system.

One of the variables that can overcome actigraphy limitation for sleep detection is wrist temperature (WT). It constitutes a good predictor for sleep onset latency (13) and sleep fragmentation (16). WT appears to anticipate light sleep by a few minutes (11). Sleep onset is anticipated and promoted by the vasodilatation of peripheral skin vessels, which causes an increase in WT (26, 27), and in consequence a drop in core body temperature. Additional increase in skin temperature was associated to phase N3. Conversely, short before and immediately after waking up, skin temperature drops, allowing core body temperature to increase. Previous validation studies using PSG, showed that WT presented the highest specificity of all variables studied (11) (motor activity, body position, and TAP), and it was also the only one whose values varied significantly with sleep phases. Thus, WT is fundamental for detecting sleep onset and offset, as well as informing on voluntary removal of the sensor. Recently, WT usefulness has been supported by a consensus document sponsored by the National Heart Lung and Blood Institute, National Institute on Aging and the Sleep Research Society (28), stating that WT constitutes a newer and less invasive method of measuring circadian phase timing and sleep and wake states.

Another source of information that eliminates the need for subjects to keep sleep diaries is the recording of exposure to light, both visible and infrared. These variables can be used for the automatic detection of the time the subject goes to bed and gets up, which makes the calculation of time in bed more accurate. For example, in the validation study, using PSG, the turning on of the infrared light source for the video recording has been used to synchronize the recording of ACM with that of PSG, which has made it possible to obtain very precise TIB estimates.

Among the device channels detecting movement and position, the variability of the position of the X axis provides a great deal of information with regard to the onset of the sleep period and changes in position in bed. This variable has recently been proposed as a source of information for the detection of sleep episodes (29), however, with this technique, the removal of the sensor would continue to be a factor of confusion in the correct detection of sleep. On the other hand, the PIM method for calculating the integrated acceleration in each time period is a good measure of the activity level and vigor of movements, while the TAT mode, which measures the time the patient remains in movement above the sensitivity threshold set on the device, constitutes an appropriate measure to evaluate the time the subject spends in an active state.

Although ACM mean values for TST, SE, and WASO were quite close to those obtained from PSG, there was a moderate degree of variability in accuracy between individuals, as evidenced by those participants with relatively large differences between ACM and PSG values particularly in WASO estimation (Figure 3). Therefore, caution should be required when considering ACM as unique method to detect sleep pathologies.

This ambulatory monitoring device meets the challenge proposed by the Movement Disorder Society Task Force on Rating Scales that suggested that the scales and questionnaires by themselves cannot adequately reflect the fluctuating nature of sleep alterations nor detect the multiple variants of sleep disorders in PD (30). This group proposes the urgent need to develop practical, specific tools to detect sleep disorders, and daytime drowsiness in large groups of PD patients. It is precisely along this line of work where we find the device evaluated in this study.

The main strengths of ACM as compared to PSG in PD can be summarized as: (a) it measures sleep parameters related to the disease in the patient's real-life environment; (b) recordings can be made over 1 or more weeks to improve the reliability and provide a broad spectrum of the subject's sleep variability; (c) greater ease in carrying out repeated evaluations over time; (d) it makes it possible to evaluate the motor function through a comparison between the TAT and PIM measurements; (e) it provides data on the evolution of the dysautonomia typical of PD through measurements of wrist skin temperature; (f) in addition, ACM provides simultaneous information on both daytime and nighttime sleep, as well as physical activity and exposure to synchronizers, such as the light-dark cycle.

In spite of all these advantages, there are some clear limitations to our study. For example, how is it possible to ensure that immobility measured by ACM corresponds to sleep episodes and how to distinguish between the bradykinesia typical of an off state from real sleep episodes? In patients with good regulation of vasomotor tone, this drawback would be avoided by adding wrist temperature to sleep detection by ACM; however, it is more complicated in the subgroup of patients with vasomotor alterations. Another limitation is the lack of a controlled diary of motor fluctuations, which limits the capacity of the observations made and renders impossible any correlation between the findings observed in the recordings and the motor and non-motor symptoms experienced by the patients.

All of the patients with PD monitored in our study showed nighttime sleep alterations, most of which have previously been described, such as poor sleep efficiency (31), lengthy nighttime movement time (32), altered thermoregulation during sleep (33), increased frequency and duration of naps (34), and a slight advance in the times for waking up and going to bed. Most of these symptoms are associated with maintenance insomnia, which is the most common sleep disorder in patients with PD (35). The etiology of insomnia in PD owes to multiple factors and includes the nighttime reemergence of motor symptoms, pain, depression, nocturia, dopaminergic medication, and the coexistence of other sleep alterations, such as sleep breathing disorders and parasomnias (1). In our study, there are no significant differences in the number of awakenings per hour in PD vs. control subjects, however, the duration of these awakenings is significantly greater in the former. It is possible that once the patient wakes up, the presence of motor and non-motor symptoms, such as anxiety (36), may make it more difficult to fall back asleep. Another possible factor is the existence in PD of nighttime akinesia, which refers to the greater limitation or difficulty in performing axial movements during sleep, such as rolling over (37, 38). In a previous study, nighttime akinesia was associated with a greater number of episodes in which the subject got out of bed due to nocturia, a common symptom in these patients (38). As shown in our recordings, patients usually turn on the bedroom light when they get up, which may be related to said nocturia, contributing to difficulty in falling asleep and prolonging awakenings.

Furthermore, even though a good correlation exists between the findings of PSG and ACM, this comparison has only been made in subjects who do not have PD. It is well known that many PD presents specific motor symptoms, with intense movements during sleep, as occurs with the REM Sleep Behavior Disorder (RBD), that can appear even in the prodromal phase of the disease. In this condition the interpretation of motor activity during sleep could differ with that for normal sleep and probably will requires of future studies in which both techniques are compared in these patients to develop. specific algorithms. However, our main objective was to validate the use of an ambulatory device (ACM, Kronowise) for the assessment of sleep-wake in wide population groups without previous selection or exclusion by pathologies or conditions. The need of specific algorithms per condition, as occurs with other commercial devices would make unviable its use for screening in huge populations when their possible pathologies are not known at priori.

In summary, the ACM KW device has proven to be clinically useful in evaluating sleep in an objective manner, thanks to the integrated management of different complementary variables, which has advantages over conventional actigraphy based on movement as a complement to PSG, although PSG continues to be the standard of reference in the diagnosis of sleep disorders. Furthermore, ACM is especially useful in those population groups that simultaneously experience autonomic, circadian and sleep alterations, as occurs in PD, making it possible to record the evolution of the disease and the development of individualized therapies to specifically improve nighttime symptoms.

## Ethics Statement

All participants received adequate information on the study and signed an informed consent form before being included. The study was approved by the Ethics Committee at the University of Murcia and HUVN.

## Author Contributions

CM-N, MR, and JM: study concept and design. FP, FE-S, and FR: acquisition of data. CM-N, FP, MC, and JM: analysis and interpretation of data. CM-N, MR, and JM: drafting of manuscript. CM-N, FP, FE-S, MC, FR, MR, and JM: critical revision.

### Conflict of Interest Statement

The authors declare that the research was conducted in the absence of any commercial or financial relationships that could be construed as a potential conflict of interest.

## Abbreviations

ACM: ambulatory circadian monitoring
AT: awake time
BT: bed time
CFI: circadian function index
GUT: gep up time
IS: interdaily stability
IV: intradaily variability
L5V and L5T: mean value and timing of the 5 consecutive hours with the lowest values
L10V and L10T: mean value and timing of the 10 consecutive hours with the lowest values
M5V and M5T: mean value and timing of the 5 consecutive hours with the highest values
M10V and M10T: mean value and timing of the 10 consecutive hours with the highest values
NT: napping time
PD: Parkinson's disease
PIM: proportional integrated mode
PLM: periodic leg movements
PSG: polysomnography
PX: position of the x axis
RA: normalized relative amplitude
SE: sleep efficiency
SL: sleep latency
TAP: intra-subject normalization of three signals, wrist skin temperature, time of movement and variability of the x-axis
TAT: time above threshold
TIB: time in bed
TM: time in movement
TMI: time in movement index
TST: total sleep time
WASO: wake after sleep onset
WT: wrist temperature.
